# PrEP Use Among Female Sex Workers: No Evidence for Risk Compensation

**DOI:** 10.1097/QAI.0000000000002134

**Published:** 2019-07-23

**Authors:** Katia Giguère, Luc Béhanzin, Fernand A. Guédou, Denis Talbot, François A. Leblond, Ella Goma-Matsétsé, Djimon M. Zannou, Dissou Affolabi, René K. Kêkê, Flore Gangbo, Moussa Bachabi, Michel Alary

**Affiliations:** a Centre de Recherche du CHU de Québec, Université Laval, Québec, Canada;; b Département de Médecine Sociale et Préventive, Université Laval, Québec, Canada;; c Currently, Department of Epidemiology, Biostatistics and Occupational Health, McGill University, Montreal, Québec, Canada;; d Dispensaire IST, Centre de Santé Communal de Cotonou 1, Cotonou, Bénin;; e Centre de Recherche de l'Hôpital Maisonneuve Rosemont, Montréal, Québec, Canada;; f Currently, Prometic Biosciences, Inc., Laval, Québec, Canada;; g Faculté des Sciences de la Santé, Université d'Abomey-Calavi, Cotonou, Bénin;; h Centre National Hospitalier Universitaire HMK de Cotonou, Cotonou, Bénin;; i Programme Santé de Lutte Contre le Sida (PSLS), Cotonou, Bénin; and; j Institut National de Santé Publique du Québec, Québec, Québec, Canada.

**Keywords:** PrEP, sexual behavior, sex workers/prostitutes, prostate-specific antigen, Y-chromosomal DNA, HIV

## Abstract

Little is known about risk compensation among female sex workers (FSW) on HIV pre-exposure prophylaxis (PrEP), and self-report of sexual behaviors is subject to bias.

**Setting:**

Prospective observational PrEP demonstration study conducted among FSW in Cotonou, Benin.

**Methods:**

Over a period of 24 months, we assessed and compared trends in unprotected sex as measured by self-report (last 2 or 14 days), by detection of sexually transmitted infections (STIs), and by vaginal detection of prostate-specific antigen and Y-chromosomal DNA, 2 biomarkers of semen exposure in the last 2 or 14 days, respectively. Trends were assessed and compared using a log-binomial regression that was simultaneously fit for all unprotected sex measures.

**Results:**

Of 255 participants, 120 (47.1%) completed their follow-up. Prevalence of STI decreased from 15.8% (95% confidence interval: 11.8% to 21.0%) at baseline to 2.1% (95% confidence interval: 0.4% to 10.2%) at 24 months of follow-up (*P*-trend = 0.04). However, we observed no trend in self-report of unprotected sex in the last 2 (*P* = 0.42) or 14 days (*P* = 0.49), nor in prostate-specific antigen (*P* = 0.53) or Y chromosomal DNA (*P* = 0.25) over the same period. We observed no statistically significant difference between trends in self-report of unprotected sex and trends in biomarkers of semen exposure in the last 2 days (*P* = 0.14) or in the last 14 days (*P* = 0.29).

**Conclusions:**

We observed no evidence of risk compensation, and a decrease in STI among FSW on PrEP. PrEP intervention may be an opportunity to control STI among FSW. Future studies should assess risk compensation with biomarkers of semen exposure when possible.

## Abstract 2

Supplemental Digital Content is Available in the Text.

## INTRODUCTION

Oral pre-exposure prophylaxis (PrEP) is an effective HIV prevention method that is strongly recommended by the World Health Organization as part of combination HIV prevention approaches for people at high risk of HIV infection.^1^ However, the use of PrEP has raised a lot of concerns about potential risk compensation: increase in HIV-related risk behaviors based on assumption of protection against HIV infection, which could lead to an increase in other sexually transmitted infections (STIs).^2^

There is no consensus about risk compensation with PrEP. A review of 18 PrEP studies conducted among people who inject drugs, serodiscordant couples, men who have sex with men and transgender women, women, and heterosexual men has shown no association between PrEP use and changes in sexual risk behaviors as measured by self-report or STI incidence.^3^ However, most of the included studies were randomized controlled trials, in which participants know they may be taking a placebo.^4^ In another review restricted to open-labelled PrEP studies among men who have sex with men, most of the 16 included studies found evidence of an increase in condomless sex as measured by self-report or STI incidence among PrEP users.^4^ Among female sex workers (FSW), one PrEP demonstration study conducted in South Africa has shown no clear change over time in self-reported consistent condom use, but a decrease in STI.^5^

A major limitation in studies assessing sexual behaviors is that self-report of sexual behaviors is subject to social desirability bias.^6–8^ In longitudinal studies, the extent of social desirability bias may also vary with repeated counselling on condom use and repeated assessment of sexual behaviors, which could in turn bias assessments of trends in self-reported sexual behaviors.^9^ STI may be considered as an objective assessment of unprotected sex; however, given that not every unprotected sex act will result in an STI, and given that a decrease in STI over time may reflect an increase in STI treatment rather than a decrease in unprotected sex, STI may not be the most valid biomarker to assess trends in unprotected sex.^7,10^ In contrast, prostate-specific antigen (PSA) and Y-chromosomal DNA (Yc-DNA), which have been shown to be valid biomarkers of recent semen exposure among women,^11–14^ are not expected to vary independently from a change in unprotected sex over the course of a study. After semen exposure, PSA and Yc-DNA can be detected in vaginal samples for up to 2 and 14 days, respectively.^11–13^ Moreover, comparison of self-reported unprotected sex in the last 2 or 14 days with PSA or Yc-DNA detection, respectively, allows assessment of under-reporting of unprotected sex over those 2 recall periods.^15–25^

Over the course of a PrEP demonstration study conducted among FSW in Cotonou, Benin, we aimed to assess potential risk compensation by evaluating trends in unprotected sex. Because we hypothesized that self-report of unprotected sex would be biased and that STI could vary independently from a change in unprotected sex, we used PSA and Yc-DNA as gold standards to detect trends in unprotected sex. To assess potential bias in trends as measured by self-report, we compared time trends in self-reported unprotected sex in the last 2 days with PSA detection, and time trends in self-reported unprotected sex in the last 14 days with Yc-DNA detection. Finally, to better define the potential bias in self-report, we assessed trends in under-reporting of unprotected sex in the last 2 or 14 days. We did not assess over-reporting of unprotected sex, because participants over-reporting unprotected sex cannot be distinguished from those who accurately report unprotected sex, but test negative for biomarkers due to the rapid decline in biomarkers' sensitivity after semen exposure.^11,12^

## METHODS

We used data from the PrEP arm of a prospective early antiretroviral therapy and PrEP demonstration study that was conducted among FSW from October 2014 to December 2016 at the Dispensaire IST (DIST) in Cotonou, Benin.^26^ To be eligible to PrEP, FSW had to be ≥18 years old, have normal renal and liver function, not have active hepatitis B, and not be pregnant or breastfeeding. Eligible FSW were recruited from October 2014 to December 2015 and followed-up on a quarterly basis until December 2016 or until a maximum of 24 months. Depending on the time of recruitment, the potential maximum length of follow-up for a participant thus varied from 12 to 24 months (administrative censorship).

At baseline, we collected sociodemographic characteristics and assessed sexual behaviors through face-to-face interviews, and we collected vaginal samples with cotton swabs. Sexual behaviors and vaginal samples were also collected every 6 months during follow-up. All interviews were administered by 2 trained staff members in a private setting at the DIST. Participants were provided monthly with daily tenofovir disoproxil fumarate/emtricitabine (Truvada; Gilead Sciences, Inc., Foster City, CA). Counselling on adherence to treatment and condom use, and free condoms, were provided in the field and at each visit at the DIST. STI treatment was provided free of charge to participants diagnosed with an STI at any time over the course of the study.

All participants provided free and informed written consent before recruitment, but the specific purpose of PSA and Yc-DNA detection was not revealed to the participants until the end of the study to limit information bias. The Benin National Ethics Committee for Health Research and the ethics committee of the CHU de Québec—Université Laval both approved the study protocol, including the procedures for delayed information.

### Self-Report of Unprotected Sex

At baseline, and at the 6-, 12-, 18-, and 24-month follow-up visits (M6, M12, M18, and M24), we interviewed the participants on their sexual behaviors from the last 2 and 14 days. For each of the 2 recall periods, we asked participants to report the number of vaginal sex acts with any type of sexual partners (clients, regular partners, and nonpaying and nonregular partners), the frequency of condom use (never, less than half of the time, at least half of the time, or always), and whether they experienced condom malfunction (breakage or slippage). Self-report of unprotected sex in the last 2 or 14 days was defined as reporting at least one vaginal sex act with any type of partner and inconsistent condom use and/or condom malfunction in the last 2 or 14 days, respectively.

### Biological Assessment of Unprotected Sex

After the interview on sexual behaviors, vaginal swabs were collected by a clinician and tested in laboratory for trichomoniasis, gonorrhea, chlamydia, PSA, and Yc-DNA. We visually assessed trichomoniasis through direct microscopy on wet mount, and we detected gonorrhea and chlamydia infections with a nucleic acid amplification test (NG/CT Probetec assay; Becton Dickenson, Cockeysville, MD) according to the manufacturer's instructions. A sample was defined as positive for STI when testing positive for at least one of the 3 assessed STIs, whereas a sample was defined as negative for STI when testing was negative for all 3 STIs.

We prepared samples for PSA and Yc-DNA detection as previously described.^25^ We tested samples for PSA with ABAcard p30, a rapid immunochromatographic assay (Abacus Diagnostics, West Hills, CA), according to the manufacturer's instructions. After 10 minutes of incubation on a strip test at room temperature, a sample was considered negative if no pink line was observed at the test (T) position, or positive if a pink line was observed at the T position. We observed no inconclusive result (ie, absence of a pink line at the control position).

We assessed Yc-DNA with a previously described nested polymerase chain reaction targeting the testis-specific protein Y-encoded family of homologous genes (Giguère et al, submitted). We amplified each sample in replicates of 3. A sample without amplification in all 3 replicates was considered negative, whereas a sample with amplification in at least one of 3 replicates was considered positive. All polymerase chain reaction products (positive controls and positive tests) were of expected size and we observed no false positive among the no template or negative controls. To avoid male DNA contamination, all laboratory procedures for the detection of Yc-DNA were performed by female technicians.

### Under-Reporting of Unprotected Sex

Under-reporting in the last 2 days was defined as having reported no unprotected sex in the last 2 days while testing positive for PSA, whereas under-reporting in the last 14 days was defined as having reported no unprotected sex in the last 14 days while testing positive for Yc-DNA.

### Statistical Analysis

We assessed unprotected sex with 5 methods: self-report in the last 2 days, self-report in the last 14 days, and STI, PSA, and Yc-DNA detection. To compare time trends in unprotected sex from baseline to M24 according to the different methods, we simultaneously fit a model for the 5 methods using a log-binomial regression. We used simultaneous modelling of the 5 methods, because contrary to individual modelling, it allows direct comparison of trends between multiple outcomes.^27^ We applied a generalized estimating equation with a log link function, an autoregressive working covariance matrix, and a binomial distribution to account for multiple observations per participant. The model included a five-level variable for the methods, a five-level visit variable (baseline, M6, M12, M18, and M24), and an interaction term between the method and visit variables to test for differences in time trends of unprotected sex as measured by the different methods. To compare trends of under-reporting of unprotected sex in the last 2 and 14 days, we fit a second model exactly as described for unprotected sex except that the five-level method variable was changed to a two-level variable for under-reporting (in the last 2 or 14 days). Linear trends and comparisons between trends were tested by contrasts.

Because of administrative censorship and withdrawals, attrition before M24 was high in our study. To limit the potential selection bias due to attrition, we repeated the previous analyses by applying 2 different strategies, both separately and in combination. The first strategy was to weight the observed data by the inverse probability of censoring to create a pseudo-population mimicking the initial cohort, that is, including the participants who were censored.^28^ The second strategy was to test trends over the first 12 months of follow-up to avoid potential selection bias due to administrative censorship. All analyses were conducted in SAS Studio, version 3.7.1 (SAS Institute, Inc., Cary, NC).

## RESULTS

### Study Population

Of the 256 FSW who were recruited in the PrEP arm of the early antiretroviral therapy/PrEP study, we excluded one participant from analyses, because she had missing data on variables that we used to calculate inverse probability-of-censoring weights (IPCW). Baseline characteristics of the 255 (99.6%) included participants are reported in Table 1. Mean age was 32.5 years (SD = 9.2), half (49.0%) of participants were Beninese, 65.9% had less than a secondary education, 97.7% were not married, 85.1% used no hormonal contraception, 98.0% had ever previously attended a condom use demonstration, 98.4% identified the condom as an effective mean to protect against HIV, 88.9% perceived themselves at risk for HIV infection, and 81.1% perceived the risk of HIV infection for a person on PrEP as being low.

**TABLE 1.**
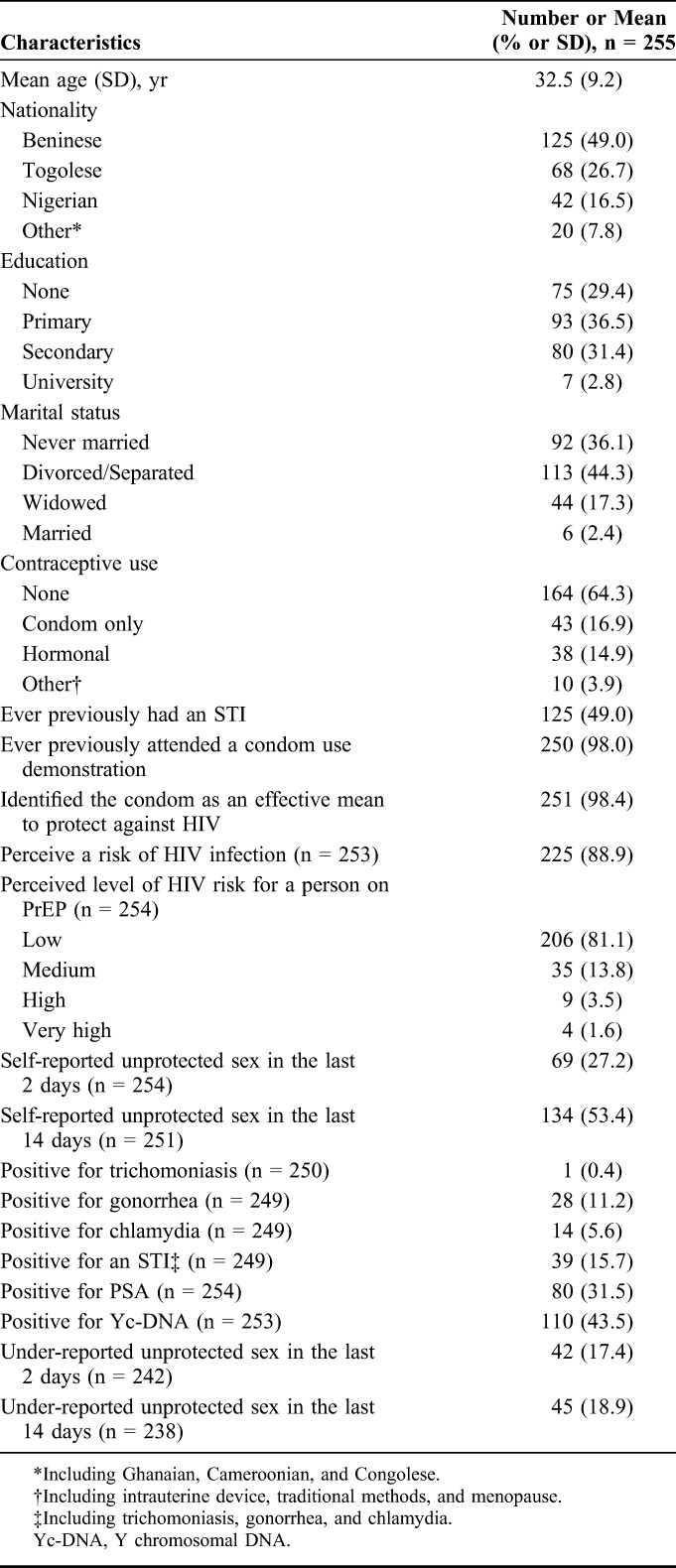
Baseline Characteristics of Female Sex Workers Participating in a PrEP Demonstration Study in Cotonou, Benin (2014–2016)

### Attrition

**FIGURE 1.**
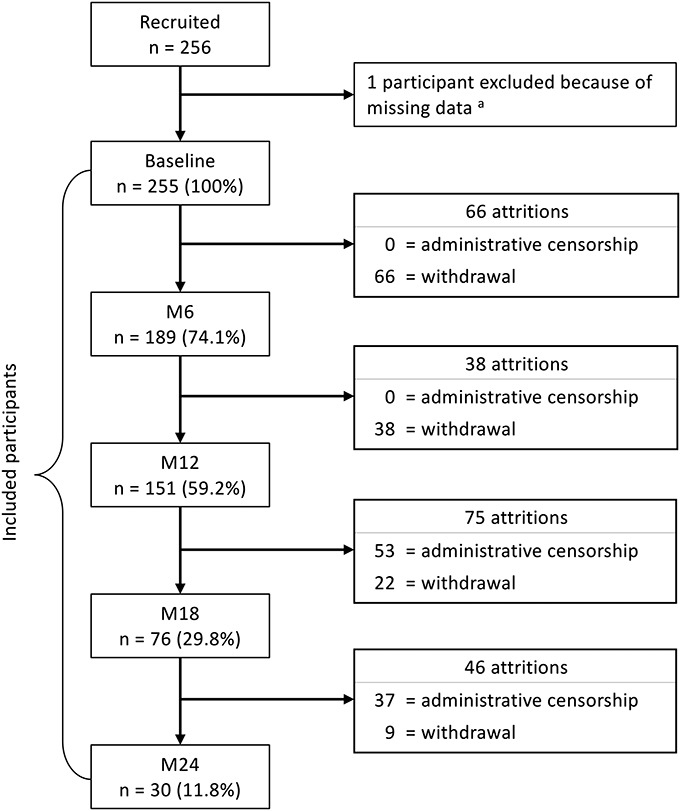
Flowchart of attrition among female sex workers participating in a PrEP demonstration study in Cotonou, Benin (2014–2016). M6, M12, M18, M24: 6-, 12- 18- 24-month follow-up visits. ^a^One PrEP participant was excluded from analyses, because she had missing data on variables that were used for the calculation of the inverse probability of censoring weights.

A total of 120 participants (47.1%) completed their follow-up (ie, did not withdraw from the study). Two hundred twenty-five (225) participants were not followed through M24 either because of administrative censorship (n = 90), or because of withdrawals (n = 135). Reasons for withdrawals are reported elsewhere.^26^ Figure 1 shows a flowchart describing attrition.

### Prevalence of Unprotected Sex and of Under-reporting of Unprotected

At baseline, 27.2% (69/254) and 53.4% (134/251) of the participants reported unprotected sex in the last 2 or 14 days, respectively (Table 1). About a sixth (15.7%, 39/249) of the participants tested positive for an STI, among which 2.6% for trichomoniasis, 35.9% for chlamydia, and 71.8% for gonorrhea. A total of 31.5% (80/254) and 43.5% (110/253) of the participants tested positive for PSA or Yc-DNA, respectively. A total of 17.4% (42/242) of the participants reported no unprotected sex in the last 2 days while testing positive for PSA, and a similar proportion (18.9%, 45/238) reported no unprotected sex in the last 14 days while testing positive for Yc-DNA (Table 1).

### Trends in Unprotected Sex and in Under-reporting of Unprotected Sex From Baseline to M24

**FIGURE 2.**
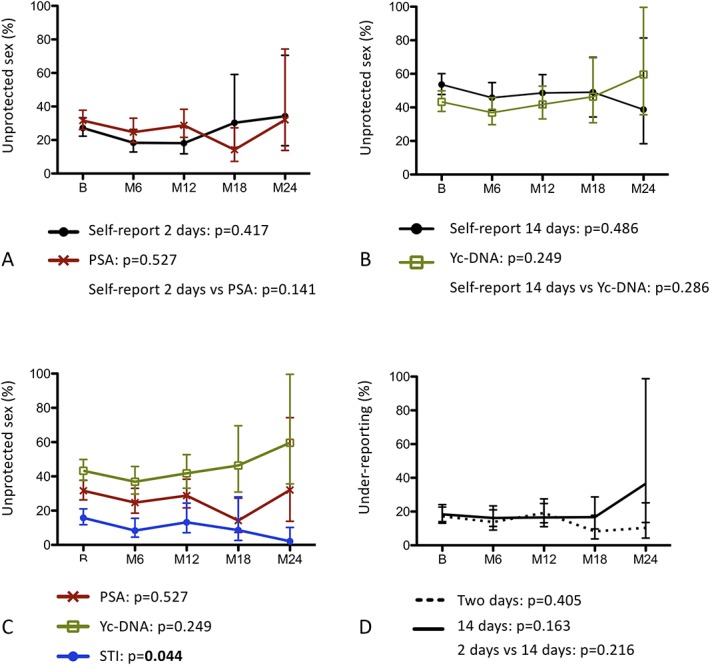
Weighted (IPCW) trends in unprotected sex and under-reporting of unprotected sex from baseline to M24 among female sex workers participating in a PrEP demonstration study in Cotonou, Benin (2014–2016). B, Baseline; M6, M12, M18, M24: 6-, 12- 18- 24-month follow-up visits; IPCW, inverse probability of censoring weighting; Yc-DNA, Y chromosomal DNA. A, Trends in unprotected sex in the last 2 days as measured by self-report or PSA. B, Trends in unprotected sex in the last 14 days as measured by self-report or Yc-DNA. C, Objective trends in unprotected sex as measured by STI, PSA, or Yc-DNA detection. D, under-reporting of unprotected sex in the last 2 days or in the last 14 days. Vertical lines denote 95% confidence intervals. Trends and comparisons between trends were assessed by contrasts: *P*-values are reported under each corresponding panel and are indicated in bold if statistically significant (*P* < 0.05).

Non-weighted (ie, without IPCW) prevalence of unprotected sex and of under-reporting according to the different detection methods are reported by visit from baseline to M24 and can be found in the Table, Supplemental Digital Content 1, http://links.lww.com/QAI/B360. From baseline to M24, self-report of unprotected sex in the last 14 days significantly decreased from 53.3% to 27.0% (*P*-trend = 0.01), but we observed no statistically significant change in Yc-DNA positivity (*P*-trend = 0.39), a biomarker of unprotected sex in the last 14 days. A statistically significant difference was observed between self-report in the last 14 days and Yc-DNA trends (*P* = 0.02), and although not statistically significant (*P* = 0.78), there was a trend toward increase in under-reporting of unprotected sex in the last 14 days (from 18.8% to 28.9%). When we applied IPCW to control for selection bias (Fig. 2 and see Table, Supplemental Digital Content 2, http://links.lww.com/QAI/B360), the trend in self-report of unprotected sex in the last 14 days was no longer statistically significant (*P* = 0.49), and there was no longer a statistically significant difference between self-report in the last 14 days and Yc-DNA trends (*P* = 0.29). We observed a statistically significant decrease in STI prevalence (*P*-trend = 0.03), and the negative trend remained statistically significant with IPCW (*P* = 0.04), with STI prevalence having decreased from 15.8% at baseline to 2.1% at M24.

### Trends in Unprotected Sex and in Under-reporting of Unprotected Sex From Baseline to M12

We also tested trends over the first 12 months of follow-up to avoid potential selection bias due to administrative censorship. Self-report of unprotected sex in the last 2 days decreased from 27.2% to 17.7% (*P* = 0.02). However, we observed no change in PSA (*P* = 0.64), a biomarker of unprotected sex in the last 2 days. Though we observed a statistically significant difference between self-report in the last 2 days and PSA trends (*P* = 0.01), we observed no trend in under-reporting of unprotected sex in the last 2 days (*P* = 0.80). After applying IPCW, the negative trend in self-report of unprotected sex in the last 2 days remained statistically significant (*P* = 0.04). From baseline to M12, STI decreased from 15.7% to 9.0% (*P* = 0.05), but the trend was not statistically significant with IPCW (*P* = 0.58).

## DISCUSSION

This study was conducted to assess potential risk compensation among FSW participating in a PrEP demonstration study in Cotonou, Benin. Because we hypothesized that self-report of unprotected sex would be biased and that STI could vary independently than a change in unprotected sex, we used PSA and Yc-DNA as gold standards to detect trends in unprotected sex. To assess potential bias in trends as measured by self-report, we compared time trends in self-reported unprotected sex in the last 2 days with PSA detection, and time trends in self-reported unprotected sex in the last 14 days with Yc-DNA detection. Finally, to better define the potential bias in self-report, we assessed trends in under-reporting of unprotected sex in the last 2 or 14 days.

From baseline to M24, we observed a statistically significant negative trend in self-report of unprotected sex in the last 14 days, but no change in Yc-DNA prevalence. Those results suggest that reporting of unprotected sex decreased after PrEP implementation, whereas the assessed behavior did not. Consistent with those results, under-reporting of unprotected sex in the last 14 days was observed at each visit and, and there was a nonstatistically significant positive trend in under-reporting of unprotected sex in the last 14 days from baseline to M24. Social desirability bias may account for under-reporting in our population. Indeed, participants may have been reluctant to report unprotected sex in a context of intensive counselling and large access to free condom supply, especially with most of them perceiving themselves at risk of HIV infection.^6,29^ It is also possible that repeated counselling on condom use have led to an increase in social desirability bias over the course of the study, explaining the observed decrease in self-report of unprotected sex in the last 14 days and increase in under-reporting in the last 14 days.^9^ However, when we corrected for potential selection bias using IPCW or when we assessed trends over the first 12 months of follow-up to eliminate potential selection bias due to administrative censorship, no trend in self-report of unprotected sex in the last 14 days and in under-reporting in the last 14 days was observed anymore, suggesting that the previously observed trends were rather due to selection bias than a real change in self-report. A possible explanation to this selection bias may be that the participants who did not withdraw or were recruited earlier in the recruitment period were participants being particularly concerned of being well-perceived and thus, tended to under-report unprotected sex to a higher extent than the participants who withdrew or were recruited later. However, this explanation should be taken with caution, because the observed positive trend in under-reporting was not statistically significant.

Noticeably, although no trend in self-report of unprotected sex in the last 2 days was observed from baseline to M24, a significant negative trend in self-report of unprotected sex in the last 2 days was observed from baseline to M12. Because of low rates of retention at M18 and M24, prevalence estimates of self-report of unprotected sex in the last 2 days were highly unstable at these follow-up visits. That is, a lack of statistical power could have prevented us from observing a negative trend in self-report of unprotected sex in the last 2 days from baseline to M24. Moreover, the trend observed in self-report of unprotected sex in the last 2 days over the first 12 months of follow-up remained significant after applying IPCW, suggesting that the proportion of participants who reported unprotected sex in the last 2 days decreased independently from a selection bias in the first 12 months of follow-up. Interestingly, no trend was observed in PSA over the same period, with or without IPCW, which suggests that unprotected sex in the last 2 days did not change from baseline to M12, but only the reporting of it. Still, an increase in social desirability bias over the course of the study due to repeated counselling on condom use might have led to a decrease in self-report of unprotected sex in the last 2 days over the first 12 months of follow-up.^9^ However, the absence of a positive trend in under-reporting in the last 2 days seems in contradiction with the combined decrease in self-report and the absence of a change in PSA. Another explanation to those results could be that our capacity to detect an increase in under-reporting was impaired by a decrease in PSA sensitivity over the course of the study. But, because we have no reason to believe that the performance of the PSA detection test decreased over the course of the study, a more likely explanation would be that there was a decrease in over-reporting of unprotected sex (ie, reporting of unprotected sex while testing negative for PSA). If true, this hypothesis would suggest that the participants tended to report sexual behaviors more accurately over time.

The absence of an increase in unprotected sex as measured by objective markers of semen exposure suggests that there was no risk compensation in our study. For risk compensation to occur, a few conditions must be met: (1) the intervention (here PrEP) must be visible to the participants, (2) the intervention must have an effect on the participants that gives rise to the perception of protection, (3) the participants must have a motivation to increase their risk-taking, and (4) the participants must have control and opportunity to adjust their behavior.^30,31^ We believe that conditions 1 and 4 were met in our study, whereas conditions 2 and 3 may have not been. Indeed, participants were aware of the intervention and, because the negotiation of condom use is a challenge for FSW,^32^ it is likely that they had full control and opportunity to abandon it if they wanted to. However, participants might not have had an increased perception of protection during the study. Indeed, for an intervention to give rise to the perception of protection, participants must not only believe in its efficacy, but they must adhere to it. In our study, most participants reported to perceive a low risk of HIV infection for a person on PrEP, but they have also shown poor adherence to PrEP, with between only 43.3% and 78.4% of participants, depending on follow-up visits, who reported having taken all pills in the previous week.^26^ Perception of protection may have not been prevalent enough in our study to translate into risk compensation at the population level. Finally, participants may have had no motivation in increasing their risk-taking. Indeed, contrary to PrEP, condoms do not only protect against HIV, but also against other STI and unwanted pregnancy. That is, even under PrEP protection, condoms still provide an additional protection that might have been perceived as being advantageous enough by the participants to avoid a decrease in condom use.

Consistent with a previous demonstration study that has shown a significant reduction in STI episodes over time among FSW on PrEP, we observed a significant decrease in STI from baseline to M24.^5^ Such a decrease in STI over time may be caused by a reduction in unprotected sex and/or an increase in STI treatment. Because no trend was observed with both PSA and Yc-DNA over the same period, the second explanation is more likely. When we applied IPCW or restricted analyses to the first 12 months of follow-up, the STI negative trend remained statistically significant. However, when we combined both strategies together to correct for selection bias, the trend in STI was no longer observed, suggesting that the observed trend may be due to selection bias. Indeed, participants who were not censored over the course of the study were actually those who had longer follow-up and thus, who received more sustained STI screening and treatment, which could explain the observed decrease in STI. Indeed, numerous studies have shown that regular STI screening and treatment is associated with a decrease in the incidence and the prevalence of STI among FSW.^33^

This study has some limitations. First, attrition was very high in our study, which led to an important decrease in statistical power over the course of the study and may have biased tests for trends. Second, although we tried to correct for the potential selection bias, we cannot exclude the possibility that this correction was incomplete. Third, PSA and Yc-DNA concentrations decline rapidly after semen exposure: previous studies have shown that about only 3%–7% of women will test positive for PSA up to 2 days after semen exposure, whereas while about only 12% of women will test positive for Yc-DNA up to 14 days after semen exposure.^11,12^ That is, prevalence of unprotected sex as measured by biomarkers, and prevalence of under-reporting of unprotected sex are likely to be underestimated in our study. This could explain, for example, that we observed lower proportions of women testing positive for Yc-DNA compared with the proportions of women reporting having had unprotected sex in the last 14 days. The lack of biomarkers' sensitivity, which does not allow to distinguish between participants who over-reported unprotected sex from participants who accurately reported unprotected sex, but falsely tested negative for biomarkers, also prevented us to study over-reporting as a potential bias in assessment of trends in self-reported unprotected sex. Nevertheless, we have no reason to believe that the lack of biomarkers' sensitivity varied over the course of the study and as such, that it would have biased assessments of risk compensation (trends in biomarkers) or of trends in under-reporting of unprotected sex. Fourth, due to the small sample size, it was not possible to assess risk compensation by stratification levels of perceived PrEP efficacy and of PrEP adherence, which may have impaired our capacity to detect risk compensation among specific subgroups of participants.^30^ Finally, our results may not be generalizable to populations that may have different perception of protection from HIV by PrEP use and different PrEP adherence levels, such as FSW in other HIV epidemic settings or women in serodiscordant couples.

This study has also important strengths. It is the first to have assessed trends in unprotected sex among women on PrEP by the means of PSA and Yc-DNA, 2 biomarkers of recent semen exposure. Contrary to self-report of sexual behaviors and to STI, PSA, and Yc-DNA are not expected to vary over time independently from a change in unprotected sex and are thus more valid measures of trends of unprotected sex, and thus of potential risk compensation in a longitudinal study. Another strength of this study is the attempt to correct for the potential selection bias due to attrition by applying IPCW and by testing trends on a shorter period of time.

## CONCLUSIONS

Our study has shown no evidence of risk compensation, and a decrease in STI prevalence after PrEP implementation among FSW in Cotonou, Benin. Though further studies are required to evaluate risk compensation and to explain its occurrence or not among FSW, our results suggest that a PrEP intervention may be a great opportunity to provide sustained STI screening and treatment for a better control of STI epidemic among this population. Noticeably, our results also suggest that bias in self-report of unprotected sex may vary over the course of a longitudinal study. Those results are concerning and point out the necessity to objectively assess trends in unprotected sex by the means of biomarkers such as PSA or Yc-DNA. Future studies should assess risk compensation with biomarkers of semen exposure, and by stratification levels of the perception of PrEP efficacy and of PrEP adherence when possible.
